# *Salmonella enterica* mediated epigenetic promotion of fibrosis is a novel factor in benign prostatic hyperplasia

**DOI:** 10.1186/s40779-025-00614-2

**Published:** 2025-05-29

**Authors:** Cong Zhu, Lu-Yao Li, Ming-Hui Shi, Cheng Fang, Lu Yang, Ting Li, Fei Li, Shi-Song Yang, Tian-Kun Wang, Dao-Jing Ming, Tong Deng, Hao-Yue Sun, Wen-Ting Li, Jia Zhang, Yu-Sen Zhang, Zhi-Yuan Jian, Chang-Jiang Qin, Shuang-Ying Wang, Xian-Tao Zeng

**Affiliations:** 1 Center for Evidence-Based and Translational Medicine, Zhongnan Hospital of Wuhan University, Wuhan, 430071 China; 2 Department of Gastrointestinal Surgery, the First Affiliated Hospital of Guilin Medical University, Guilin, 541001 Guangxi China; 3 Department of Urology, Zhongnan Hospital of Wuhan University, Wuhan, 430071 China; 4 Department of Gastrointestinal Surgery, Huaihe Hospital of Henan University, Kaifeng, 475000 Henan China; 5 Department of Urology, West China Hospital, Sichuan University, Chengdu, 610041 China; 6 Department of Thyroid and Breast Surgery, Zhongnan Hospital of Wuhan University, Wuhan, 430071 China

**Keywords:** Benign prostatic hyperplasia (BPH), *Salmonella enterica* (*S. enterica*), Fibrosis, Epigenetic, AlkB homolog 5 (ALKBH5)

## Abstract

**Background:**

Fibrosis constitutes a significant pathophysiological mechanism in the clinical progression of benign prostatic hyperplasia (BPH) and represents a contributing factor to the ineffectiveness of prevailing pharmacological treatments. Emerging evidence suggests a close association between microbial presence and the development of fibrosis. Nonetheless, the potential involvement of microbes within prostatic tissue in the pathogenesis of BPH and prostatic fibrosis, along with the underlying mechanisms, remains unexplored.

**Methods:**

Utilizing immunohistochemistry and microbial sequencing, we analyzed the microbes of prostate tissues from BPH patients with different degrees of prostate fibrosis and found that *Salmonella enterica* (*S. enterica*) was enriched in the high degree of prostate fibrosis. We developed prostate cell and animal models infected with the lipopolysaccharide of *S. enterica* (*S.e*-LPS) to assess its impact on prostate fibrosis. To elucidate the underlying functional mechanisms, we employed molecular biology techniques, including RNA degradation assays, N^6^-methyladenosine (m^6^A) dot blotting, RNA immunoprecipitation, and m^6^A immunoprecipitation.

**Results:**

Microbial diversity differed between low- and high-fibrosis groups, with *S. enterica* showing the highest mean abundance among the four species that differed significantly. *S.e*-LPS was detected in *S. enterica*-rich prostate tissue and was found to significantly promote cell proliferation, cell contractility, lipid peroxidation, and the induction of ferroptosis. Animal experiments demonstrated that *S.e*-LPS infection led to pronounced hyperplasia of the prostatic epithelium, with epithelial thickness increasing to 1.57 times that of the sham group, and collagen fibrosis increasing to 2.84 times that of the sham group, thereby exacerbating prostatic tissue fibrosis in rats. In vitro experiments further revealed that *S.e*-LPS promoted prostate cell fibrosis by inducing ferroptosis. Mechanistically, it was determined that *S.e*-LPS regulates ferroptosis via AlkB homolog 5 (ALKBH5)-mediated m^6^A modification, which affects the stability of glutathione peroxidase 4 (*GPX4*) mRNA, thereby affecting prostatic fibrosis.

**Conclusion:**

The findings of this study suggest that *S. enterica* promotes prostatic fibrosis through ALKBH5-m^6^A-GPX4-mediated ferroptosis. This research offers novel insights for the development of new therapeutic targets and personalized strategies for the prevention and treatment of BPH from the perspectives of microbes and epigenetics.

**Supplementary Information:**

The online version contains supplementary material available at 10.1186/s40779-025-00614-2.

## Background

Benign prostatic hyperplasia (BPH) is a common age-related disease in men, typically emerging after the age of 40, with incidence rates reaching approximately 50% and 90% at ages 60 and 90, respectively [1]. Clinically, BPH presents with lower urinary tract symptoms and is pathologically characterized by non-malignant hyperproliferation of epithelial and stromal cells within the prostate’s transition zone. Research has shown that collagen deposition and myofibroblast accumulation play an important role in the development of BPH [2]. Sheng et al. [3] identified that early-progressed BPH, necessitating surgical intervention before the age of 50, is a fibrosis-related condition marked by excessive collagen deposition. Prostatic fibrosis has recently been recognized as a significant pathobiological process contributing to the accelerated clinical progression of BPH and is implicated in the ineffectiveness of conventional pharmacological treatments, such as 5α-reductase inhibitors and α1-adrenergic receptor antagonists [4]. Nonetheless, research exploring the underlying mechanisms of prostatic fibrosis remains limited.

Tissue fibrosis is thought to be influenced by genetic factors, microbial invasion, inflammation, lifestyle choices, external stimuli, and aging [5]. Evidence indicates a significant association between microbial presence and the development of fibrosis. For instance, *Staphylococcus aureus* has been shown to induce mammary gland fibrosis in mice by activating the Toll-like receptors (TLR)/nuclear factor kappa-B (NF-κB) and TLR/activator protein 1 (AP-1) signaling pathways [6], while *Cryptococcus gattii* infection induces pulmonary inflammation and fibrosis [7]. Our previous research identified the presence of the oral-specific bacterium *Porphyromonas gingivalis* in prostate fluid, which significantly induced collagen deposition in a rat model of bacterial infection [8]. However, the potential role of the microbes in prostate tissue in the context of BPH and prostatic fibrosis has not been investigated. *Salmonella enterica* (*S. enterica*), a major pathogen responsible for foodborne illnesses, is known to cause a range of gastrointestinal and systemic diseases in humans [9]. Ehrhardt et al. [10] showed that *S. enterica* infection in mice can induce intestinal fibrosis through the regulation of proteases and protease inhibitors, mirroring the pathology observed in human diseases. However, the contribution of *S. enterica* to the progression of BPH or its potential to induce prostatic fibrosis remains to be elucidated.

N^6^-methyladenosine (m^6^A) is recognized as the most prevalent and abundant post-transcriptional modification, influencing a wide range of biological processes [11]. Liu et al. [12] identified 92,046 hypermethylated CpG sites and 10,117 hypomethylated CpG sites in different genomic regions of BPH through epigenetic analysis, and found a negative correlation between promoter methylation and gene expression, indicating that DNA methylation serves as a key mechanism of transcriptional regulation in BPH. Furthermore, Li et al. [13] demonstrated that methyltransferase-like (METTL) 3 facilitates BPH progression by modulating phosphatase and tensin homolog deleted on chromosome ten (PTEN) expression in an m^6^A-YTH domain family protein (YTHDF) 2-dependent manner. Accumulating evidence suggests that microbial entities can influence host epigenetic mechanisms. For instance, *Fusobacterium nucleatum* significantly reduces m^6^A modification in colorectal cancer cells and tissues by down-regulating the m^6^A methyltransferase METTL3, thereby accelerating colorectal cancer aggressiveness and metastasis [14]. Additionally, intracellular infection by *Mycobacterium tuberculosis* triggers the phosphorylation of METTL14 and subsequent liquid phase separation, which impairs the host’s anti-tuberculosis immune response [15]. However, the impact of microbial influence on m^6^A modification within the prostate and its role in BPH progression has not been studied, as well as the role and underlying mechanism remain to be elucidated.

In this study, we aim to focus on the therapeutic challenge of prostatic fibrosis in BPH. Through microbial sequencing of different fibrotic prostate tissues from BPH surgical patients, we aim to elucidate the potential relationship between microorganisms and prostatic fibrosis. Furthermore, we aim to investigate the effects of *S. enterica* on the biological function of prostate cells, ferroptosis, and prostatic fibrosis through cellular, animal, and molecular biology experiments, and clarify its potential mechanism on prostatic fibrosis, providing new insights and evidence for the prevention and treatment of BPH.

## Methods

### Participants and study design

BPH patients (*n* = 19) were recruited from Zhongnan Hospital of Wuhan University between November 2019 and October 2020. Eligible participants were those diagnosed with BPH who underwent transurethral resection of the prostate (TURP). Exclusion criteria encompassed patients with concurrent urogenital malignancies, urinary tract infections, or postoperative pathology reports indicating conditions other than BPH. Patient electronic medical records and clinical laboratory test results of the patients were collected for subsequent analysis. This study protocol was approved by the Ethics Committee of Zhongnan Hospital of Wuhan University (2,019,102).

### Disease diagnosis and sample collection

BPH diagnosis was conducted by experienced physicians according to the patient’s medical history, the International Prostate Symptom Score (IPSS), and clinical examination results. Approximately 8–10 g of prostatic tissue resected during TURP were collected, thoroughly washed with normal saline, and immediately placed into sterile cryotubes. These samples were then preserved in liquid nitrogen for subsequent analyses. The entire procedure, encompassing both the surgical intervention and sample collection, was performed under strict aseptic conditions.

### Microbial DNA extraction, sequencing and analysis

Microbial genomic DNA was extracted from prostate tissue using MagPure Stool DNA KF kit B (Magen, China) according to the manufacturer’s instructions. Then, a reaction system was configured with qualified DNA and specific primers (V3V4 region, 338F: ACTCCTACGGGAGGCAGCAG, 806R: GGACTACHVGGGTWTCTAAT), and the PCR products were purified by magnetic beads, followed by sequencing. Data were preprocessed using FLASH (v1.2.11), USEARCH (v7.0.1090), and UCHIME (v4.2.40) to obtain Operational Taxonomic Units (OTUs) of clusters, as detailed in our previous study [16]. OTU annotation was performed by the RDP classifier (v2.2). α diversity analysis and principal coordinate analysis (PCoA) analysis were performed using vegan (v.2.6.8) and ape (v.5.8) R packages, and partial least squares discriminatory analysis (PLS-DA) was performed using mixOmics (v.6.28.0) R package [17]. Microbial Kyoto Encyclopedia of Genes and Genomes (KEGG) function prediction was performed using phylogenetic investigation of communities by reconstruction of unobserved states (PICRUSt2) 2 software (v2.2.0-b) [18]. The Wilcox test package in R (v4.4) was used to analyze microbial and functional differences.

### Animal experiments

Animal studies were performed in strict accordance with the protocol approved by the Animal Ethics Committee of Zhongnan Hospital of Wuhan University (20,240,286), and compliant with the ARRIVE guidelines. All rats in this experiment were raised in the Animal Experiment Center of Zhongnan Hospital of Wuhan University, and their drinking water and feed were sterilized by autoclaving. All rats were adaptively fed for one week before the experiment and kept in the same living environment and food during the experiment to eliminate confounding factors such as diet, obesity, and stress. Data analysis personnel were blinded to the group assignments of the animals. No animals or data were excluded.

*For experimental design 1* Twenty-four 7-week-old male Sprague-Dawley rats [body weight: (258.25 ± 11.15) g were used in this animal experiment. The rats were randomly divided into 4 groups (*n* = 6/group): 1) Sham group (Sham) was injected with 100 μl PBS with equal amounts into the right and left ventral lobes of the prostate; 2) *S.e*-LPS group (*S.e*-LPS) were injected with 100 μl *S.e*-LPS (100 μg/kg, L6143, Sigma, St. Louis, MO, USA) with equal amounts into the right and left ventral lobes of prostate; 3) Testosterone-induced BPH (T-BPH) group underwent castration and 100 μl PBS was injected into the prostate, and subcutaneous injection of testosterone propionate [5 mg/(kg·d)] for 4 weeks [19–21]; 4) Testosterone-induced BPH group underwent castration and *S.e*-LPS (100 μl, 100 μg/kg) was injected into the prostate (T-BPH + *S.e*-LPS), and subcutaneous injection of testosterone propionate [5 mg/(kg·d)] for 4 weeks. All rats received subcutaneous injections in the week after surgery. The above surgical procedures were conducted under anesthesia by intraperitoneal injection of sodium pentobarbital (40 mg/kg).

*For experimental design 2* Eight 7-week-old male Sprague-Dawley rats [body weight: (286.62 ± 30.10) g were used in this animal experiment. For AlkB homolog 5 (*ALKBH5*) knockdown in rat prostate, shRNA and corresponding control lentiviruses were synthesized by GeneChem (Shanghai, China). The rats were divided into 2 groups (*n* = 4/group). All testosterone-induced BPH rats (T-BPH) underwent castration and subcutaneous injection of testosterone propionate [5 mg/(kg·d)] for 2 weeks. Then, shALKBH5 and shNC groups were injected with 5 μl corresponding lentivirus (4 × 10^8^ TU/ml) with equal amounts into the right and left ventral lobes of the prostate. Then, testosterone propionate [5 mg/(kg·d)] was administered subcutaneously for 2 weeks. The above surgical procedures were conducted under anesthesia by intraperitoneal injection of sodium pentobarbital (40 mg/kg). The ALKBH5 shRNA target sequence was shown in Additional file 1: Table S1.

Following the completion of the experiment, all rats were weighed and euthanized using an overdose of anesthetic. The bladder, seminal vesicles, and prostate were meticulously excised, with the capsule and adipose tissue stripped, and then photographed and weighed. The prostate weight index was calculated as [prostate wet weight per animal (mg)/body weight per animal (g)] × 100%. The prostate tissues were subsequently fixed with 4% paraformaldehyde fixative (Beijing Labgic Technology Co., Ltd., Beijing, China) for subsequent histological examination. The H&E and Masson’s trichrome stainings of rat prostate tissues were analogous to those used for human prostate tissues. Histopathological staining was analyzed using ImageJ software as previously described [20].

### Hematoxylin–eosin (H&E), Masson’s trichrome, and Immunohistochemistry (IHC) staining

The paraffin-embedded prostate tissue blocks were sectioned into 4 μm thick slices using a pathology microtome (Shanghai Leica Instrument Co., Ltd, RM2016). Following dewaxing and rehydration, the sections underwent staining with an H&E HD constant dye kit (G1076, Servicebio, Wuhan, China) and a Masson dye solution set (G1006, Servicebio, Wuhan, China) according to the manufacturer’s instructions. After clearing and sealing, the slides were photographed using an orthostatic light microscope (Nikon Eclipse E100, Japan). The area percentages of collagen fibers (blue) and muscle fibers (red) in Masson-stained images were quantitatively analyzed using ImageJ software (National Institutes of Health, USA) [22]. The samples were categorized into two groups: low-fibrosis (LF, *n* = 9) and high-fibrosis (HF, *n* = 10), based on the median collagen fiber content of 20.5%. For IHC staining, 1% hydrogen peroxide was used to block endogenous peroxidase activity. The corresponding primary antibody was incubated at 4 °C overnight, and subsequently incubated with the secondary antibody at room temperature for 30 min. After using the DAB chromogenic agent, dehydrate the tissue, and use a neutral gum chip. All antibodies are shown in the Additional file 1: Table S2.

### Cell culture and cell transfection

WPMY-1 cell lines were cultured in DMEM medium (Gibco, Carlsbad, USA) containing 5% fetal bovine serum (FBS; Gibco, USA), and BPH-1 cell lines were cultured in 1640 medium (Gibco, Carlsbad, USA) containing 10% fetal bovine serum (FBS; Gibco, USA), 1% penicillin and streptomycin (NCM Biotech, China) at 37 °C with 5% CO_2_. All cell lines used in the study are free of mycoplasma. In this study, commercially available lipopolysaccharide from *S. enterica* (*S.e*-LPS, L6143, Sigma, St. Louis, MO, USA) was diluted with PBS to a concentration of 1 µg/ml for cell treatment. For *ALKBH5* knockdown, shRNA and corresponding control lentiviruses were synthesized by GeneChem (Shanghai, China), and infected cells were selected using puromycin (2 μg/ml; Biosharp, Anhui, China). ALKBH5 shRNA target sequences were shown in Additional file 1: Table S1.

### RNA extraction and reverse transcription-quantitative polymerase chain reaction (RT-qPCR) analysis

Total RNA was extracted and reversed into cDNA using TRIzol reagent and HiScript Q RT SuperMix for qPCR Kit (Vazyme, Nanjing, China). The primer sequence used in the experiment is shown in Additional file 1: Table S3. Then, using ChamQ Universal SYBR qPCR Master Mix Kit (Vazyme, Nanjing, China) to measure relative mRNA expression levels, and 2^−ΔΔCt^ is used to count the expression level relative to β-actin. Detailed experimental procedures were performed as described previously [23].

### Western blotting

Protein samples were prepared using cell lysis buffer for Western and IP (Beyotime, China) and SDS-PAGE gels. Proteins were transferred onto PVDF membranes (Millipore, USA) and blocked with 5% BSA for 1 h at 25 °C. The membranes were incubated with the appropriate primary antibody at 4 °C overnight, followed by incubation with the secondary antibody at 25 °C for 1 h. The relative expression level of protein was calculated using a chemiluminescence imaging system. All antibodies are shown in the Additional file 1: Table S4.

### m^6^A dot blot assay

Total RNA, obtained from cells and tissues, was denatured at 70 °C for 10 min, and subsequently, samples were immediately placed on ice for a minimum of 2 min and then aliquoted into subgroups containing 200, 400, and 800 ng of RNA. An equal volume of RNA samples was loaded onto a nylon membrane (Beyotime, Shanghai, China). Then, put the nylon membrane in an 80 °C drying oven for 2 h to bind the RNA to the nylon membrane. Nylon membrane block and antibody incubation refer to Western blotting. The results were also detected by Western blotting.

### RNA immunoprecipitation (RIP) assays

RIP assay was performed in WPMY-1 cells using the EZ-Magna RIP™ RNA-Binding Protein Immunoprecipitation Kit (Millipore, USA) following the manufacturer’s instructions. In brief, magnetic beads pre-coated with 5 μg normal antibodies against ALKBH5 (Merk, GER) or rabbit IgG (Millipore, USA) were incubated with cell lysates (1 × 10^7^ cells per sample) at 4 °C overnight. Then, the beads containing immunoprecipitated RNA-protein complexes were treated with proteinase K (10 mg/ml) to degrade proteins. Subsequently, RNAs were purified, and the expression of glutathione peroxidase (*GPX*) 4 in immunoprecipitated RNAs was evaluated using RT-qPCR.

### Methylated RNA immunoprecipitation (MeRIP) assays

To quantify the m^6^A levels in *GPX4* mRNA, the MeRIP assay was conducted following previously established protocols [23]. Experiments were performed using the MeRIP Kit (Epigentek, USA). Five micrograms of anti-m^6^A antibody (Epigentek, USA) or normal rabbit IgG was bound to magnetic beads (Millipore, USA). A total of 50 μg RNA extracted from WPMY-1 cells was subjected to immunoprecipitation using the anti-m^6^A antibody in RIP immunoprecipitation buffer (Millipore, USA) at 4 °C overnight. After treatment with proteinase K (10 mg/ml), RNAs were purified, and the expression of *GPX4* in immunoprecipitated RNAs was assessed by RT-qPCR assay. Specific primer sequences were employed to detect *GPX4* methylation modification levels via qPCR [24].

### RNA degradation assay

WPMY-1 cells (1.5 × 10^5^ cells/well) from the different groups were seeded in 6-well plates. Actinomycin-D (MCE, HY-17559) was added to a final concentration of 5 μmol/L, and cells were collected before or 1, 2, 3, and 4 h after adding actinomycin-D. The RNA of the cells was then extracted for RT-qPCR, and the data were normalized to the t = 0 time point.

### Statistical analysis

Statistical analyses were performed with the SPSS software version 26.0 (SPSS, Inc., Chicago, IL, USA). Gene expression, cell cycle, apoptosis, and lipid peroxidation data were analyzed using the two-tailed Student’s *t*-test. The cell proliferation curves and IC_50_ curves were evaluated using a two-way analysis of variance (ANOVA). IC_50_ curves and values were generated using GraphPad Prism 8.0. Data were expressed as mean ± standard deviation (SD). *P* < 0.05 was taken as statistically significant.

## Results

### *S. enterica* associated with prostate fibrosis

**Fig. 1:**
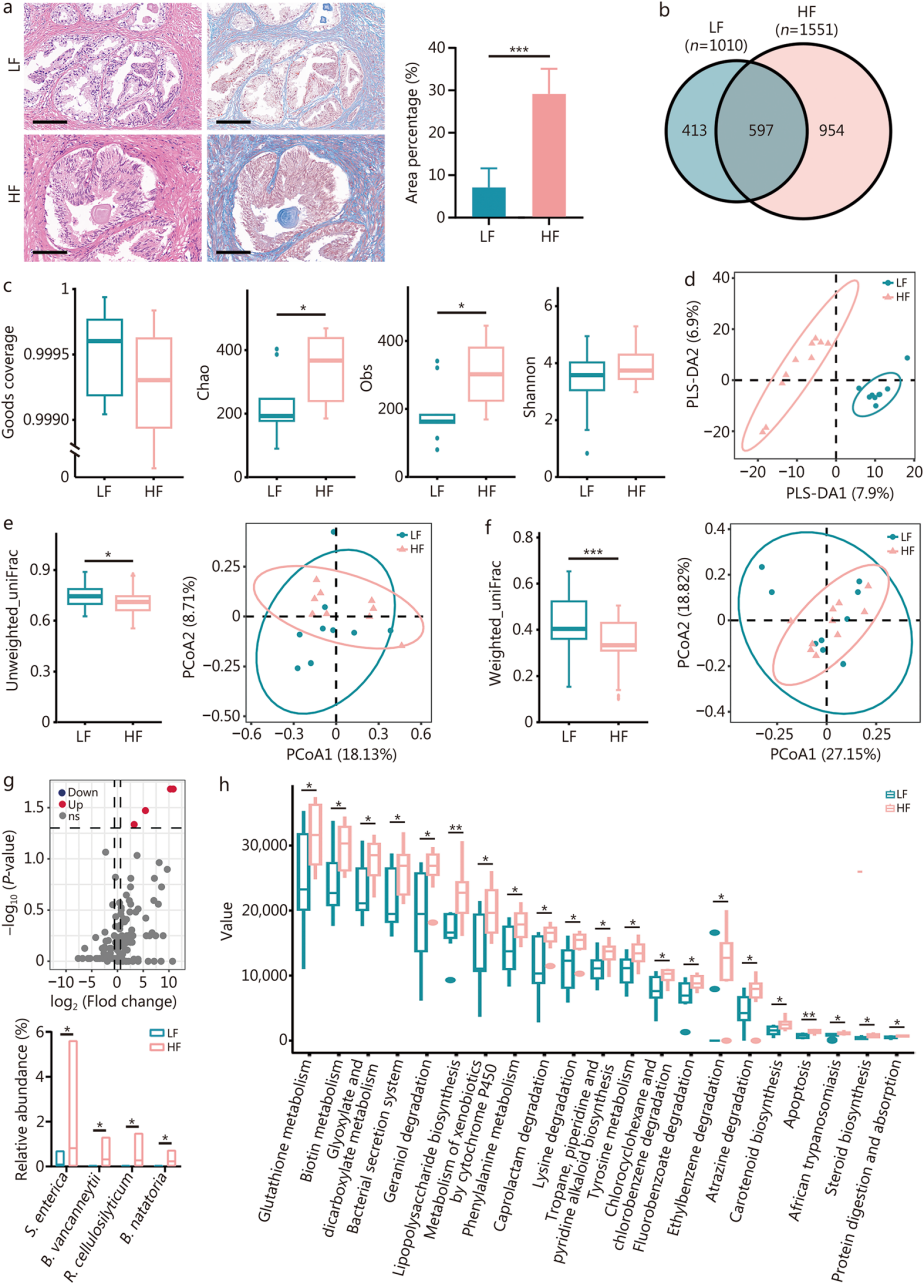
*S. enterica* was significantly enriched in BPH patients with high fibrosis. **a** Collagen fiber content detected by Masson staining in LF and HF groups. Scale bar = 100 μm. **b** The Venn diagram of the number of OUTs in the LF and HF groups. **c** The diversity analysis of Goods coverage, Chao, Obs, and Shannon index in LF and HF groups. **d** PLS-DA analysis of LF and HF groups. The β diversity analysis of PCoA based on Unweighted_uniFrac (**e**) and Weighted_uniFrac (**f**) distance in LF and HF groups. **g** Volcano plot of bacterial differences between LF and HF groups. Bar graph of significantly different bacteria in the LF and HF groups. **h** Differentially enriched KEGG functions pathways (level 3) between the two groups by PICRUSt2 analysis. ^*^P < 0.05, ^**^P < 0.01, ^***^P < 0.001. LF low-fibrosis group, HF high-fibrosis group, OTUs operational taxonomic units, PLS-DA partial least squares discriminant analysis, PCoA principal coordinate analysis, *S. entierica Salmonella enterica*, *B. vancanneytii Brevundimonas vancanneytii*, *R. cellulosilyticum Rhizobium cellulosilyticum*, *B. natatoria Blastomonas natatorial*, KEGG Kyoto Encyclopedia of Genes and Genomes, PICRUSt2 phylogenetic investigation of communities by reconstruction of unobserved states 2

Nineteen BPH patients were categorized into LF and HF groups based on the severity of fibrosis, with no significant differences in clinical parameters except for higher IPSS scores in the HF group (Additional file 1: Table S5). There was a significant difference in the number of collagen fibers detected in the prostate tissues between the LF and HF groups (Fig. 1a). A total of 1964 OTUs were detected by 16S microbial sequencing, with 597 OTUs common to both groups (Fig. 1b). The two sample groups exhibited a coverage rate over 99% with no statistically significant difference, while α diversity metrics (Chao and Obs) showed significant differences, and the Shannon index increased in HF without significance (Fig. 1c). The PLS-DA analysis, PCoA analysis based on unweighted_uniFrac, and weighted_uniFrac distance all showed that the bacterial community composition differed significantly between the LF and HF groups (Fig. 1d-f). Volcanic map analysis revealed 4 bacteria that were significantly up-regulated in the HF group compared with the LF group, with *S. enterica* exhibiting the highest mean abundance (Fig. 1g). In addition, microbial function prediction analysis showed that pathways associated with glutathione metabolism, biotin metabolism, glyoxylate, and dicarboxylate metabolism, lipopolysaccharide biosynthesis, tyrosine metabolism and apoptosis in the HF group were significantly higher than those in the LF group (Fig. 1h). The above results suggested that intra-tissue *S. enterica* may contribute to in the progression of BPH and prostatic fibrosis, potentially through glutathione metabolism related functional pathways.

### *S.e*-LPS promotes prostatic hyperplasia and fibrosis in rats

**Fig. 2:**
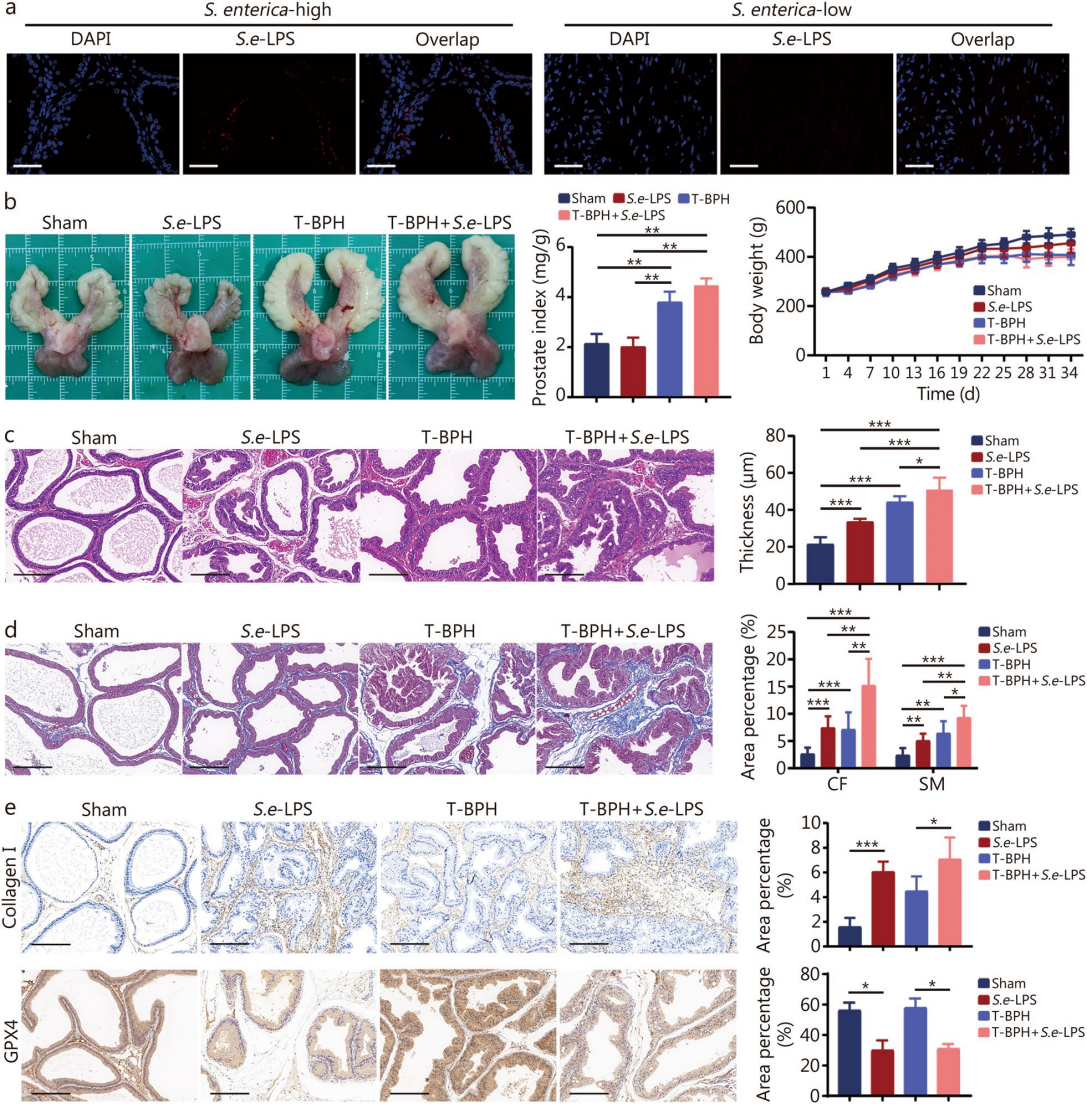
*S.e*-LPS promotes the development of BPH and tissue fibrosis in rat prostate tissue. **a** Representative image of *S.e*-LPS in prostate tissue detected by immunofluorescence; red represents *S.e*-LPS and blue represents nuclei. Scale bar = 50 μm.** b** Photographs of rat prostate tissue from the healthy, sham, *S.e*-LPS, T-BPH, and T-BPH + *S.e*-LPS groups, and histogram of prostate weight index (prostate weight/body weight) × 1000 and line plot of body weight of rats in the four groups. **c** Representative figures from H&E staining and bar graph for the area percentage of epithelia from the four groups. Scale bar = 200 μm. **d** Representative figures from Masson staining and bar graph for area percentage of SM and CF from the four groups. Scale bar = 200 μm. **e** Representative images and quantitative analysis histograms of collagen I and GPX4 immunohistochemical staining in prostate tissue of rats in the four groups. Scale bar = 200 μm. ^*^P < 0.05, ^**^P < 0.01, ^***^P < 0.001. BPH benign prostatic hyperplasia, *S. enterica Salmonella enterica*, *S.e*-LPS lipopolysaccharide of *Salmonella enterica*, DAPI 4′,6-diamidino-2-phenylindole, T-BPH testosterone-induced BPH, H&E hematoxylin–eosin, SM smooth muscle, CF collagen fibers, GPX4 glutathione peroxidase 4

*S.e*-LPS was detected in *S. enterica*-rich prostate tissue (Fig. 2a). To investigate the potential role of *S. enterica* in promoting prostatic fibrosis, we injected *S.e*-LPS into rat prostate tissues and subsequently examined the morphological and histopathological alterations. The results showed that *S.e*-LPS did not affect the gross observation of prostate tissue or the prostate index in rats compared with the sham group, and there was no significant difference in body weight gain among the 4 experimental groups (Fig. 2b). However, the H&E staining results of rat prostate tissue showed that *S.e-*LPS induced notable histomorphological changes in the prostate. In the sham group, the prostate tissue structure appeared normal, with acini containing homogeneous eosinophilic substances. The epithelial thickness in the *S.e*-LPS, T-BPH, and T-BPH + *S.e*-LPS groups was 1.57-, 2.07-, and 2.38- fold greater than that of the sham group, respectively. The epithelial thickness of the T-BPH + *S.e*-LPS group is 1.52 times that of the *S.e*-LPS group. The thickness and size of the prostate connective tissue increased in the *S.e*-LPS group and the epithelial cell layer and lumen space expanded (Fig. 2c). Masson staining further demonstrated a marked increase in stromal cells within the prostate of *S.e*-LPS-treated rats. Both the collagen fibers (CF) and smooth muscle (SM) exhibited an increase in the *S.e*-LPS, T-BPH, and T-BPH + *S.e*-LPS groups, with CF areas being 2.84-, 2.72-, and 5.80- fold, and SM areas being 2.11-, 2.68-, 3.88- fold greater than those in the sham group, respectively. Moreover, the CF and SM areas of the T-BPH + *S.e*-LPS group are 2.05-, 1.84-times that of the *S.e*-LPS group, respectively (Fig. 2d). The T-BPH + *S.e*-LPS group demonstrated the most pronounced increase in epithelial hyperplasia, SM, and CF (*P* < 0.05; Fig. 2c, d). Immunohistochemical analysis revealed a significant increase in collagen I deposition in the prostate of *S.e*-LPS rats, with the number of collagen I positive cells in the *S.e*-LPS group (3.81-fold) was significantly higher than that in the sham group, and T-BPH + *S.e*-LPS group (1.58-fold) was significantly higher than that in T-BPH group (Fig. 2e). While in response to *S.e*-LPS exposure, the content of GPX4 exhibited a decreasing trend. The number of GPX4 positive cells was significantly reduced in the *S.e*-LPS group (0.53-fold) compared with the sham group, and the T-BPH + *S.e*-LPS group (0.54-fold) showed a significant reduction compared with T-BPH group (Fig. 2e). The above results of prostate histopathological changes, indicated that *S.e*-LPS could induce prostatic fibrosis and exacerbate BPH.

### *S.e*-LPS promotes prostatic fibrosis through ferroptosis

**Fig. 3:**
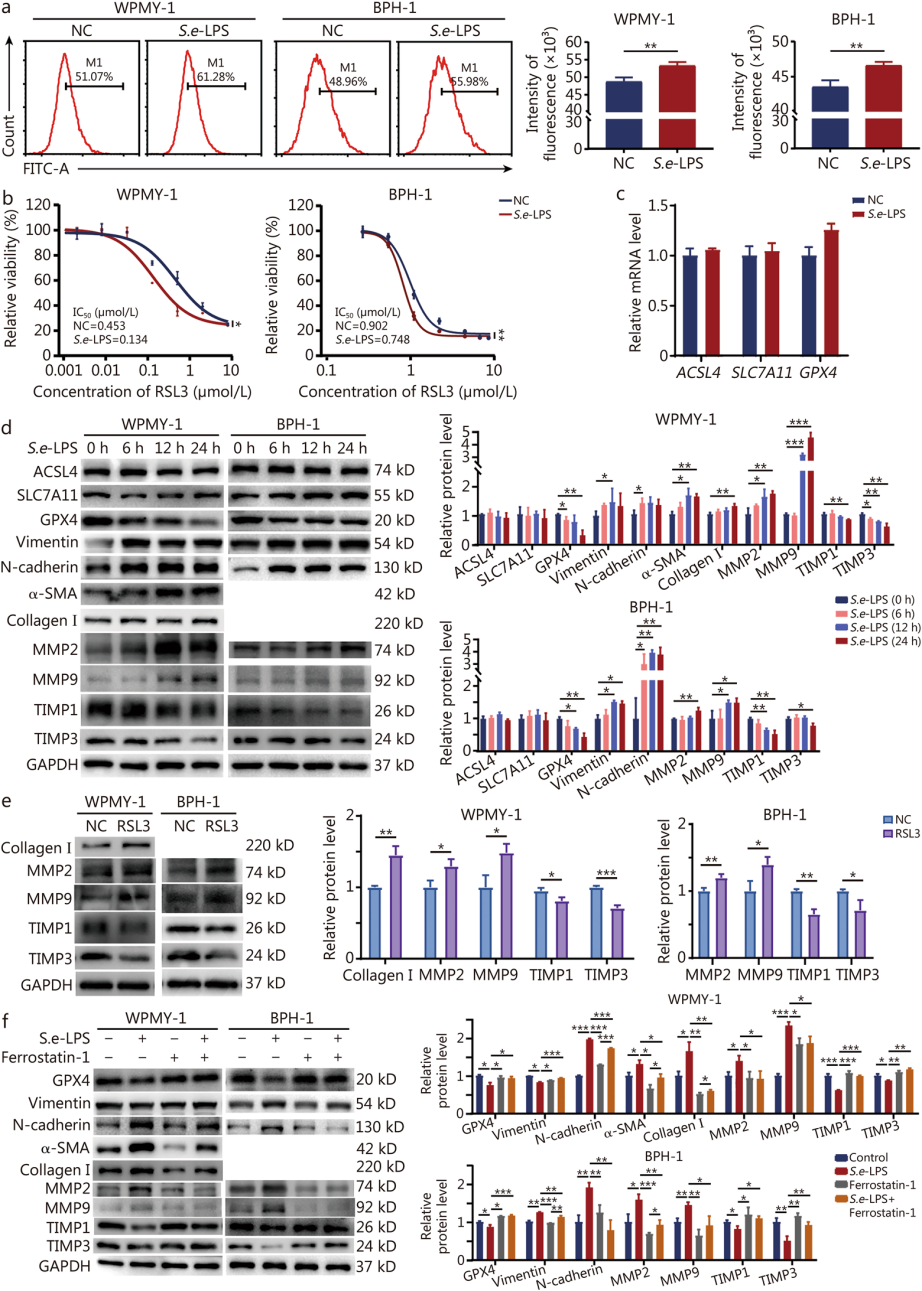
*S.e*-LPS promotes ferroptosis and fibrosis in prostate cells. **a** Flow representative images and quantitative analysis of lipid peroxidation index C11 after treating with *S.e*-LPS for 24 h in WPMY-1 and BPH-1 cells. **b** Prostate cell lines were treated with RSL3 for 24 h in the presence of PBS or *S.e*-LPS. **c** The mRNA expression of the ferroptosis gene *GPX4*, *SLC7A11* and *ACSL4* in BPH-1 cells after treating with *S.e*-LPS. **d** Expression of ferroptosis and fibrosis-related proteins in response to *S.e*-LPS stimulation at different time points of WPMY-1 and BPH-1 cells. **e** Expression of ferroptosis and fibrosis-related proteins were detected by Western blotting in prostate cell lines treated with RSL3. **f** Expression of ferroptosis and fibrosis-related proteins in the presence of *S.e*-LPS, Ferrostatin-1, or *S.e*-LPS + Ferrostatin-1. ^*^P < 0.05, ^**^P < 0.01, ^***^P < 0.001. *S.e*-LPS lipopolysaccharide of *Salmonella enterica*, RSL3 ras-selective lethal 3, GPX4 glutathione peroxidase 4, SLC7A11 solute carrier family 7 member 11, ACSL4 acyl-coa synthetase long-chain family member 4, α-SMA α-smooth muscle actin, MMP matrix metalloproteinase, TIMP tissue inhibitor of metalloproteinases

To explore the role of *S. enterica* in BPH, we examined the functional effects of *S.e-*LPS on prostate cells. The results showed that *S.e-*LPS significantly promoted the proliferation of WPMY-1 and BPH-1 cells (Additional file 1: Fig. S1a). *S.e-*LPS could enhance the contractility of WPMY-1 cells by 9.9 times (Additional file 1: Fig. S1b). However, *S.e-*LPS had no significant effect on the cell apoptosis or cycle in WPMY-1 and BPH-1 cells (Additional file 1: Fig. S1c, d). Ferroptosis, a form of cell death characterized by lipid peroxidation, was assessed using C11, a fluorescent dye for detecting lipid peroxide formation. Lipid peroxidation was significantly increased in the prostate cell line following *S.e-*LPS treatment (Fig. 3a). At the same time, reactive oxygen species (ROS) production was also significantly increased (Additional file 1: Fig. S1e). Transmission electron microscopy revealed morphological changes in mitochondria, including reduced size, increased membrane density, decreased or absent cristae, and disrupted outer membranes (Additional file 1: Fig. S1f). The levels of malondialdehyde (MDA), a product of lipid oxidation, were significantly increased by *S.e*-LPS (Additional file 1: Fig. S1g). *S.e-*LPS significantly increased the sensitivity of prostate cells to Ras-selective lethal (RSL) 3, a classical ferroptosis inducer (Fig. 3b). The above experiments collectively confirm that *S. enterica* can induce ferroptosis in prostate cells. These findings suggest that ferroptosis in prostate cells may be a causative factor for fibrosis.

Subsequent analysis of ferroptosis-related gene mRNA levels revealed that *S.e-*LPS did not alter the mRNA levels of acyl-coa synthetase long-chain family member 4 (*ACSL4*), solute carrier family 7 member 11 (*SLC7A11*), and *GPX4* (Fig. 3c). Similarly, *S.e-*LPS did not significantly affect the protein levels of ACSL4 and SLC7A11. However, *S.e-*LPS significantly decreased the expression of GPX4, tissue inhibitor of metalloproteinases (TIMP) 1, and TIMP3, while increasing the expression of Vimentin, N-cadherin, α-smooth muscle actin (α-SMA), collagen I, matrix metalloproteinase (MMP) 2 and MMP9 in prostate cells, with the effects on GPX4, α-SMA, collagen I, MMP2, MMP9, and TIMP3 being time-dependent (Fig. 3d). Upon administering the ferroptosis inducer RSL3 to prostate cells, we observed a significantly upregulation of the classical fibrosis markers collagen I, MMP2 and MMP9, alongside a notable downregulation of TIMP1 and TIMP3 (Fig. 3e). To further investigate the involvement of ferroptosis in *S.e-*LPS-induced prostatic fibrosis, we co-treated prostate cells with the ferroptosis inhibitor Ferrostatin-1 and *S.e-*LPS. The results showed that Ferrostatin-1 could reverse *S.e-*LPS-induced increase in fibrotic protein expression, including Vimentin, N-cadherin, MMP2, MMP9, collagen I and α-SMA, as well as the decrease in TIMP1 and TIMP3 expression (Fig. 3f). These results suggest that *S. enterica* can promote ferroptosis and fibrosis in prostate cells, and may regulate fibrosis through ferroptosis.

### ***S.e***-LPS regulates m^6^A levels in prostate cells via ALKBH5

**Fig. 4:**
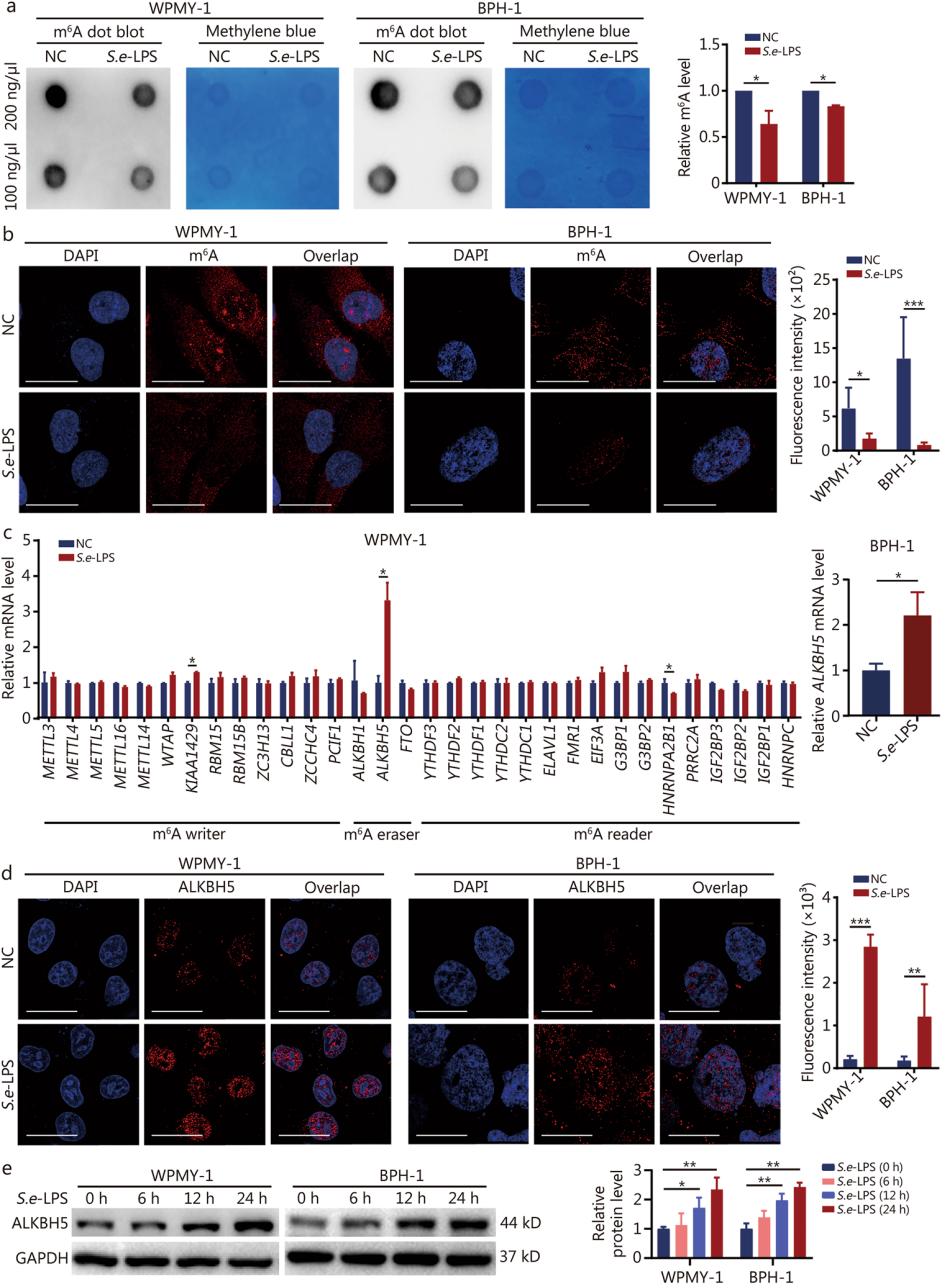
*S.e*-LPS regulates m^6^A levels in prostate cells via ALKBH5. **a** Dot blot results were obtained in WPMY-1 and BPH-1. **b** Immunofluorescence representative images and quantitative analysis of WPMY-1 and BPH-1 cells, with red representing m^6^A and blue representing the nucleus. Scale bar = 20 μm. **c** The mRNA expression levels of m^6^A-related genes in prostate cells after *S.e*-LPS treatment. **d** Immunofluorescence representative images and quantitative analysis of WPMY-1 and BPH-1 cells, with red representing ALKBH5 and blue representing the nucleus. Scale bar = 20 μm. **e** Western blotting analysis of ALKBH5 protein expression in response to S.e-LPS stimulation at different time points of WPMY-1 and BPH-1 cells. *P < 0.05, ^**^P < 0.01, ^***^P < 0.001. NC negative control, *S.e*-LPS lipopolysaccharide of *Salmonella enterica*, m^6^A N^6^-methyladenosine, METTL methyltransferase like, WTAP Wilms tumor 1-associated protein, RBM15 RNA binding motif protein 15, ZC3H13 zinc finger CCCH-type containing 13, CBLL1 Casitas B-lineage lymphoma proto-oncogene-like 1, ZCCHC4 zinc finger, CCHC-type containing 4, PCIF1 phosphorylated CTD interacting factor 1, ALKBH AlkB homolog, FTO fat mass, and obesity-associated protein, YTHDF YTH domain family member, YTHDC2 YTH domain containing 2, ELAVL1 ELAV-like RNA binding protein 1, FMR1 fragile X mental retardation 1, EIF3A eukaryotic translation initiation factor 3 subunit A, G3BP GTPase activating protein binding protein, HNRNPA2B1 heterogeneous nuclear ribonucleoprotein A2/B1, PRRC2A proline-rich coiled-coil 2A, IGF2BP insulin-like growth factor 2 mRNA binding protein HNRNPC heterogeneous nuclear ribonucleoprotein C

The above research results indicate that *S.e*-LPS significantly downregulated the protein level of GPX4, a marker of cellular ferroptosis, without significantly affecting its RNA expression level. This result suggests that *S.e*-LPS might influence the translational process of GPX4. m^6^A, a common RNA modification, has been implicated in the regulation of translation, as well as in the modulation of ferroptosis and fibrosis [24, 25]. Therefore, we speculate that m^6^A may play a pivotal role in the regulation of GPX4 expression induced by *S. enterica*. Dot blot assay results showed that *S.e-*LPS significantly reduced the cellular m^6^A levels in prostate cells (Fig. 4a). Meanwhile, cellular immunofluorescence (IF) results further demonstrated that *S.e-*LPS markedly decreased m^6^A levels in prostate cells (Fig. 4b). The m^6^A modification is a dynamically reversible co-transcriptional process. “Writers” like METTL3 and METTL14 add m^6^A marks, “erasers” such as fat mass and obesity-associated protein (FTO), ALKBH5 remove them, and “readers” including YTHDF1, YTHDC1 recognize the marks, mediating functional outcomes [26]. FTO and ALKBH5 are the main demethylases for RNA, and ALKBH1 demethylates DNA and RNA. m^6^A readers like YTHDC1 bind m^6^A-modified RNA, affecting translation, stability, and metabolism [27]. We examined these m^6^A-related genes in response to *S.e-*LPS in WPMY-1 cells using RT-qPCR. The results demonstrated a significant increase in *ALKBH5* expression, which was similarly observed in BPH-1 cells (Fig. 4c). As an m^6^A demethylase, ALKBH5 was predominantly localized in the nucleus. Immunofluorescence showed that the expression of ALKBH5 significantly increased after *S.e-*LPS treatment (Fig. 4d). Meanwhile, the protein expression of ALKBH5 significantly increased in a time-dependent manner under *S.e-*LPS treatment (Fig. 4e). These results suggest that *S.e-*LPS may participate in the progression of BPH via ALKBH5-mediated m^6^A modification.

### Knockdown of *ALKBH5* inhibits ferroptosis and fibrosis in vitro and in vivo

**Fig. 5:**
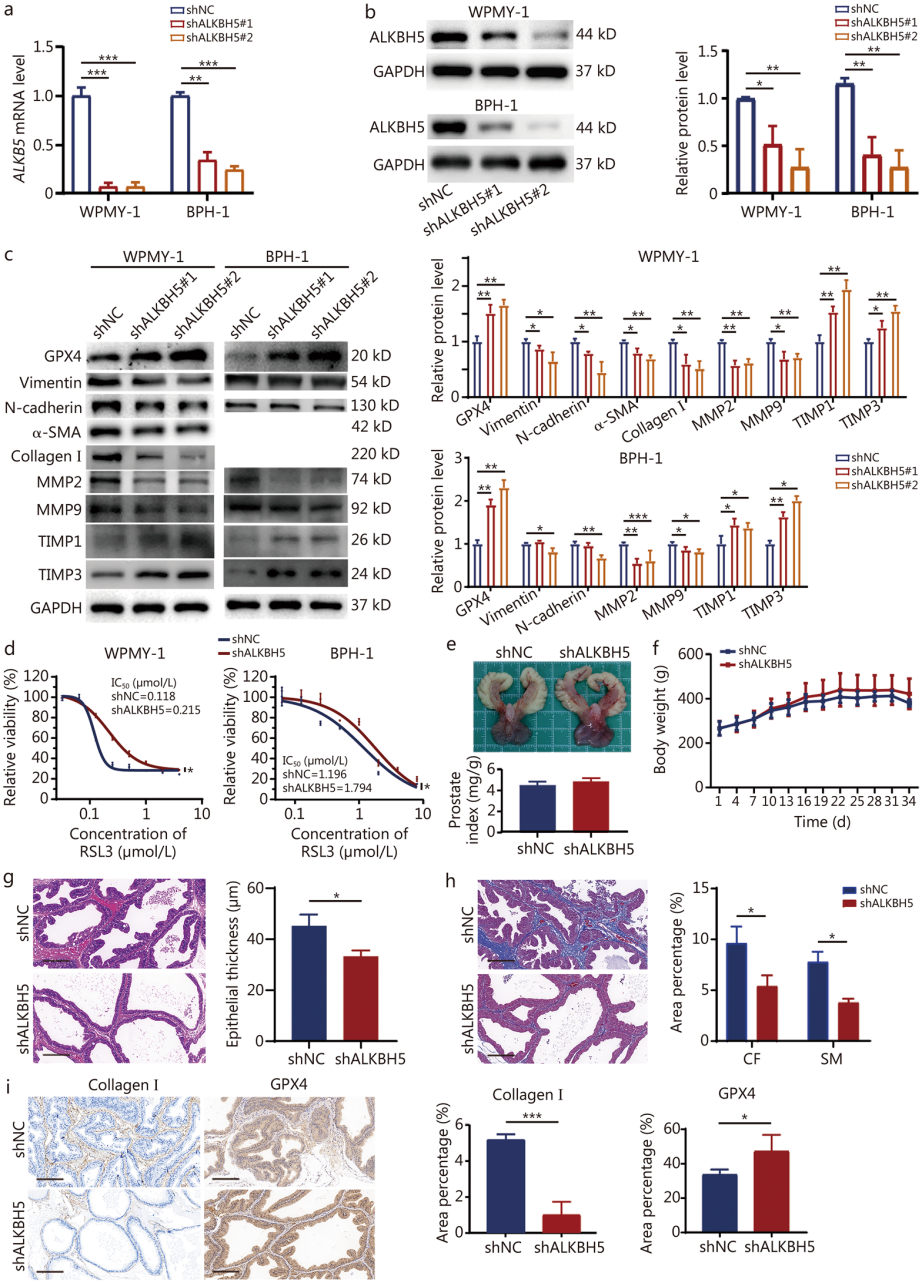
Knockdown of *ALKBH5* inhibits ferroptosis and fibrosis in prostate cells. **a** Knockdown of *ALKBH5* was assessed by RT-qPCR. **b** Western blotting showed that ALKBH5 protein was knocked down. **c** Western blotting analysis of the expression of ferroptosis and fibrosis-related proteins after *ALKBH5* protein was knocked down. **d** Prostate cell lines were treated with RSL3 for 24 h after ALKBH5 protein was knocked down. **e** Photographs of rat prostate tissues and histograms of the prostate weight index [prostate wet weight (mg)/body weight (g)] × 100% in the shNC and shALKBH5 groups. **f** The body weight growth curves of rats in the shNC and shALKBH5 groups over different days. Representative images and quantitative analysis of the HE staining (**g**), Masson’s staining (**h**), and immunohistochemical staining of (**i**) GPX4 and collagen I for the prostate tissues. Scale bar = 200 μm. ^*^P < 0.05, ^**^P < 0.01, ^***^P < 0.001. GPX4 glutathione peroxidase 4, α-SMA α-smooth muscle actin, MMP matrix metalloproteinase, TIMP tissue inhibitor of metalloproteinases, RSL3 ras-selective lethal 3, H&E hematoxylin and eosin, SM smooth muscle, CF collagen fibers, ALKBH5 AlkB homolog 5

To further investigate the role of ALKBH5 in regulating ferroptosis and fibrosis in prostate cells, we generated *ALKBH5* knockdown cell lines. Subsequent qPCR and Western blotting analyses confirmed a significant reduction in ALKBH5 expression in these cells (Fig. 5a, b). Following *ALKBH5* knockdown, the protein expression of GPX4, TIMP1, and TIMP3 in prostate cells significantly increased, while the protein expression of Vimentin, N-cadherin, α-SMA, collagen I, MMP2, and MMP9 significantly decreased. In BPH-1 cells, the protein expression of Vimentin and N-cadherin significantly decreased only in one interference sequence (Fig. 5c). At the same time, the sensitivity of prostate cells to the ferroptosis inducer RSL3 was decreased, indicating increased resistance (Fig. 5d).

Then, *ALKBH5* knockdown model was established by injecting lentivirus targeting ALKBH5 into rat prostate tissue. The results showed that *ALKBH5* knockdown did not influence the prostate index in rats (Fig. 5e), and there was no significant difference in body weight between the experimental and control groups (Fig. 5f). H&E staining revealed that following *ALKBH5* knockdown, there were significant morphological alterations in the prostate connective tissue, including a reduction in tissue volume, a decrease in the number of epithelial cell layers, and a diminished lumen size. Quantitative histological analysis showed that the epithelial layer in the shALKBH5 group was 0.74-fold compared with the shNC group (Fig. 5g). Masson’s trichrome staining demonstrated a marked decrease in stromal cells within the prostate of *ALKBH5* knockdown rats. Quantitative analysis of CF and SM revealed that the CF area was reduced to 0.56-fold, and the SM area to 0.49-fold, compared with the shNC group, respectively (Fig. 5h). Immunohistochemical analysis revealed that *ALKBH5* knockdown in rats resulted in a decrease in collagen I levels and a significant increase in GPX4 expression within the prostate tissue. The proportion of collagen I positive cells in the shALKBH5 group was lower than that of the shNC group by 0.19-fold, whereas the percentage of GPX4 positive cells in the shALKBH5 group was 1.41-fold higher than that in the shNC group (Fig. 5i).

### *S.e*-LPS regulates* GPX4* mRNA stability through ALKBH5 to promote ferroptosis and fibrosis in prostate cells

**Fig. 6:**
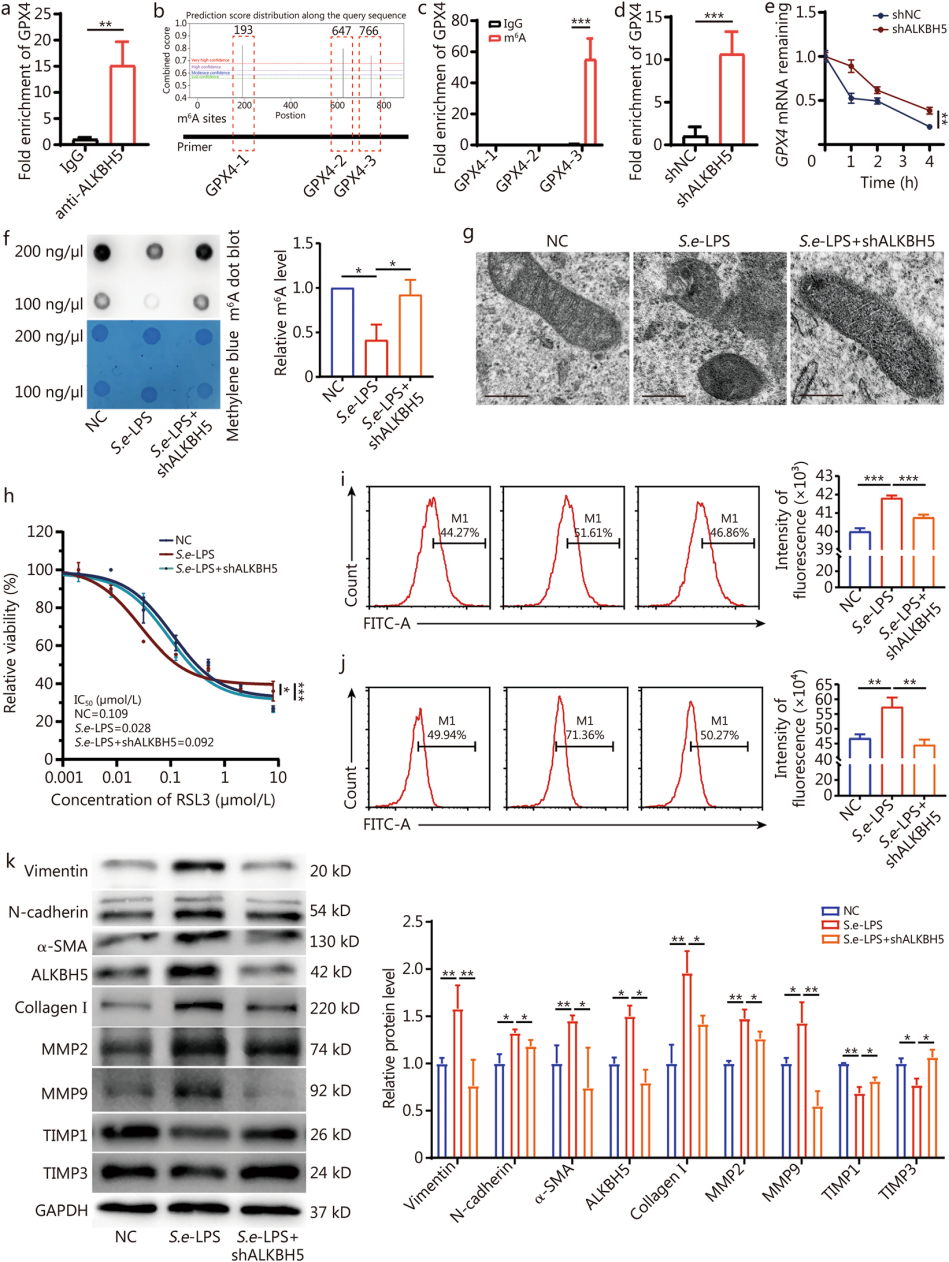
*S.e*-LPS promotes ferroptosis and fibrosis of prostate cells by regulating the stability of *GPX4* mRNA through ALKBH5.** a** The binding effects of ALKBH5 to GPX4 mRNA were detected by RIP assay in WPMY-1 cells. **b** The modification sites were predicted by SRAMP, and the three pairs of primers were designed based on the predicted m^6^A site. **c** MeRIP-qPCR were detected after *ALKBH5* knockdown. **d** The binding effects of ALKBH5 to *GPX4* mRNA were detected by MeRIP assay after the knockdown of *ALKBH5* in WPMY-1 cells. **e** The stability of *GPX4* mRNA relative to time 0 h was measured by RT-qPCR after blocking new RNA synthesis with actinomycin D (10 μg/ml) in WPMY-1 cells knockdown *ALKBH5* and control. **f** Dot blot of WPMY-1 cells in the presence of *S.e*-LPS and *S.e*-LPS combined with knockdown of *ALKBH5*. **g** Representative images of transmission electron microscopy of WPMY-1 cells. Scale bar = 200 nm. **h** RLS3 treatment was conducted to clarify the rescue effect of ALKBH5 on the WPMY-1 cells. Results of flow cytometry of lipid peroxidation marker C11 (**i**) and ROS (**j**). **k** Western blotting analysis was used to detect the levels of fibrosis-related proteins, confirming the rescuing effect of shALKBH5 on *S.e*-LPS-induced fibrosis in WPMY-1 cells. ^*^P < 0.05, ^**^P < 0.01, ^***^P < 0.001. SRAMP sequence-based RNA adenosine methylation site predictor, *S.e*-LPS lipopolysaccharide of *Salmonella enterica*, GPX4 glutathione peroxidase 4, α-SMA α-smooth muscle actin, MMP matrix metalloproteinase, TIMP tissue inhibitor of metalloproteinases, ALKBH5 AlkB homolog 5

To clarify whether *S.e-*LPS-induced upregulation of ALKBH5 modulates GPX4 expression via m^6^A methylation to trigger ferroptosis, we conducted a RIP assay, which confirmed that *GPX4* mRNA is recognized and bounded by ALKBH5 (Fig. 6a). By using sequence-based RNA adenosine methylation site predictor (SRAMP) prediction program (http://www.cuilab.cn/sramp), a tool for predicting mammalian m^6^A sites, we identified that *GPX4* mRNA can undergo m^6^A modification, with 3 high confidence methylation sites located at positions 193, 647, and 766 (Fig. 6b). Then we designed primers targeting these specific sites, and subsequent MeRIP-qPCR analysis confirmed that m^6^A modification occurs at position 766 on *GPX4* mRNA (Fig. 6c, d). To assess whether ALKBH5 influences the stability of *GPX4* mRNA, Actinomycin D (ActD) was used to block new RNA synthesis, subsequently, RT-qPCR was employed to determine the degradation rate of *GPX4* mRNA. We found that the knockdown of *ALKHB5* significantly promoted the stability of *GPX4* mRNA in WPMY-1 cells (Fig. 6e). Thus, mechanistically, ALKBH5 regulates the stability of its target gene *GPX4* mRNA.

To determine whether ALKBH5 mediates *S.e-*LPS-induced ferroptosis and fibrosis in prostate cells, we constructed a cell model treated with both *S.e-*LPS and ALKBH5 interfering lentivirus. Functionally, knockdown of *ALKBH5* rescued the *S.e-*LPS-induced reduction in m^6^A levels (Fig. 6f). Additionally, *ALKBH5* knockdown ameliorated *S.e-*LPS induced mitochondrial damage (Fig. 6g) as well as partially rescued the increased sensitivity of WPMY-1 cells to RSL3, which was induced by *S.e-*LPS (Fig. 6h). Knockdown of *ALKBH5* partially attenuated the *S.e-*LPS-induced lipid peroxidation and ROS production (Fig. 6i, j). Additionally, we found that the downregulation of GPX4, TIMP1, and TIMP3 induced by *S.e*-LPS were partially reversed after *ALKBH5* knockdown, while the upregulation of Vimentin, N-cadherin, α-SMA, collagen I, MMP2, and MMP9 induced by *S.e*-LPS were also partially reversed (Fig. 6k). Taken together, these findings indicate that *S.e-*LPS promotes ferroptosis and fibrosis in prostate cells through regulating *GPX4* mRNA via ALKBH5, thereby contributing to the progression of BPH.

## Discussion

**Fig. 7:**
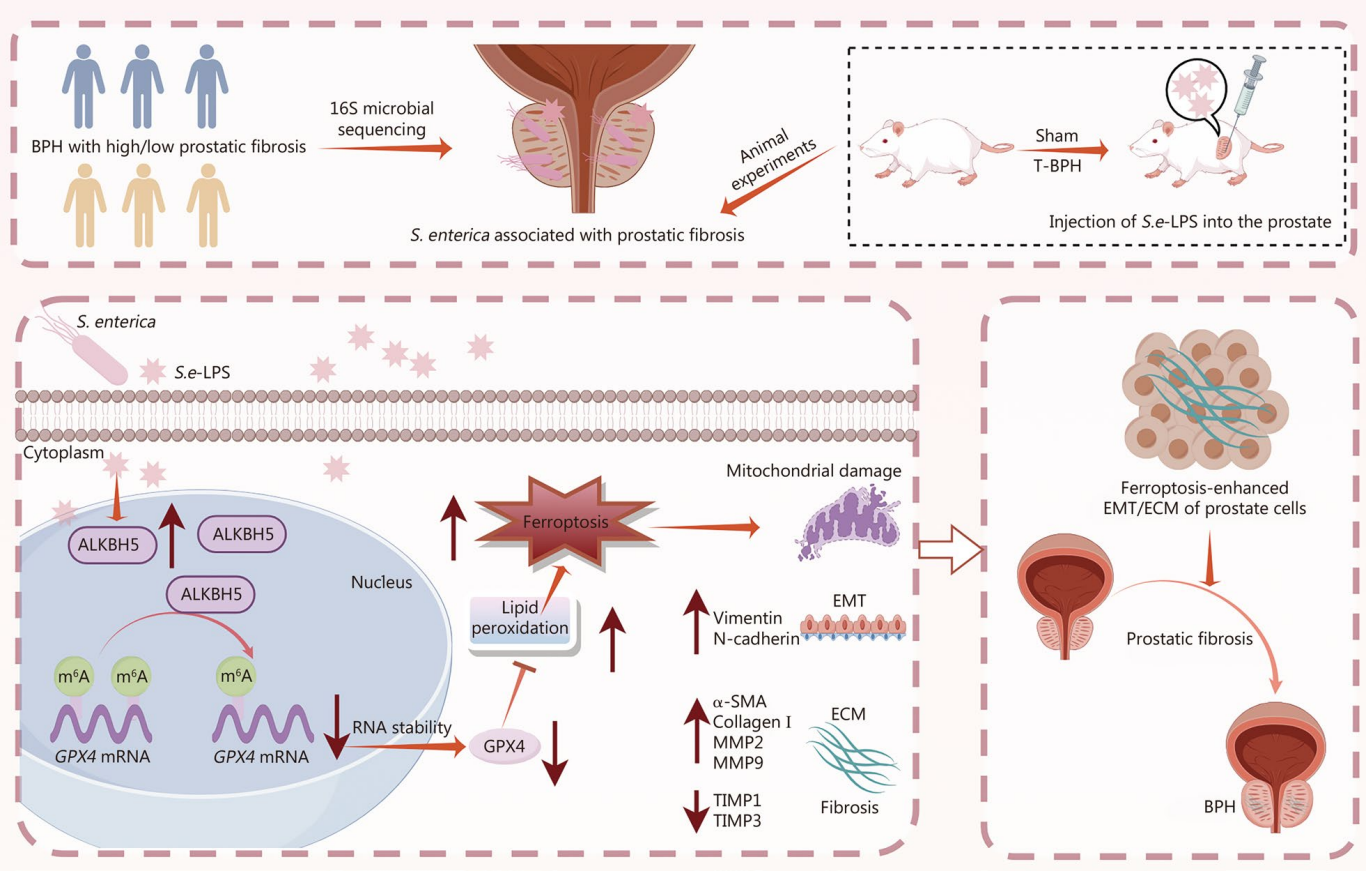
Schematic diagram of the role and mechanism of *S. enterica* in prostatic fibrosis and BPH. BPH benign prostatic hyperplasia, *S. enterica Salmonella enterica*, *S.e*-LPS lipopolysaccharide of *Salmonella enterica*, ECM extracellular matrix, EMT epithelial-mesenchymal transition, GPX4 glutathione peroxidase 4, Vim vimentin, N-cad N-cadherin, α-SMA α-smooth muscle actin, MMP matrix metalloproteinase, TIMP tissue inhibitor of metalloproteinases, ALKBH5 AlkB homolog 5

Microbes may participate in the progression of BPH through various mechanisms. In hyperplastic prostatic tissues, sustained chronic inflammation facilitates prostate fibrosis and hyperplasia by promoting extracellular matrix (ECM) deposition [28]. This pathological cascade may be mechanistically associated with inflammation-induced epithelial-mesenchymal transition (EMT), myofibroblast differentiation, ECM accumulation, and increased tissue stiffness [29, 30]. This study focused on the pathological process of prostate fibrosis for the first time and analyzed the microbial community characteristics in prostate tissues with varying degrees of fibrosis. Okada et al. [31] demonstrated through comparative analysis of the microbiota in prostate tissue and catheterized urine that the microbiota of prostate tissue is not a contaminant of the urinary microbiota, and the microbial diversity in the benign prostate enlargement (BPE) group was significantly lower than in the non-BPE group. Our study yielded similar results, showing a reduced α diversity of the microbiota in patients with higher prostatic fibrosis. In addition, *S. enterica* was identified in prostate tissues, with its abundance notably higher in tissues exhibiting advanced fibrosis. Subsequent cellular and animal experiments utilizing *S.e*-LPS, the virulence factor of *S. enterica*, confirmed that *S. enterica* can promote prostate fibrosis both in vivo and in vitro. Mechanistically, *S. enterica* affects the stability of *GPX4* mRNA through ALKBH5-mediated m^6^A epigenetic modification, which regulates ferroptosis and subsequently impacts the fibrosis process of prostate tissue (Fig. 7).

The epigenetic regulation of m^6^A mRNA modification is implicated in numerous physiological and pathological processes. Our results suggest, for the first time, that intra-tissue *S. enterica* can contribute to the progression of prostate fibrosis and induce BPH through ALKBH5-mediated m^6^A regulation of GPX4. By comparing the m^6^A status of germ-free mice with that of specific pathogen-free mice, Wang et al. [32] found that the presence of microorganisms significantly reduces the m^6^A levels in brain and intestinal tissues, which was consistent with our results. Rana et al. [33] also observed that following *Salmonella* infection, the demethylase KDM6B is upregulated and the overall host H3K27me3 level was decreased. These findings further suggest that *Salmonella* can modulate epigenetic mechanisms to exert its effects. A previous study has shown that ALKBH5 expression is significantly decreased in brain and intestinal tissues in the presence of microorganisms [32]. Another study reported that while *Fusobacterium nucleatum* does not affect ALKBH5 expression, it promotes colorectal metastasis by reducing METTL3-mediated m^6^A levels [14]. Additionally, Jabs et al. [34] found that gut microbiota, *Akkermansia muciniphila*, and *Lactobacillus plantarum* affected the level of m^6^A in the liver, without significantly affecting ALKBH5 expression. The specific m^6^A writers (such as METTL3) or erasers (such as FTO and ALKBH5) that microbes utilize to exert their effects may vary depending on the microbial species and the tissue location.

Ferroptosis is defined as a lipid peroxidation-driven and iron-dependent programmed cell death that is the result of an imbalance between cellular metabolism and redox homeostasis. This process is implicated in various physiological mechanisms and diseases. Udensi et al. [35] demonstrated that oxidative stress and abnormal elevations in ROS excess are mechanisms contributing to the development of BPH. Zhou et al. [36] reported that the activity of antioxidant enzymes was lower and MDA was higher in the prostate of rats with testosterone propionate-induced BPH, and the consumption of the antioxidant green tea polyphenol epigallocatechin‑3‑gallate could inhibit the oxidative damage of the prostate and reduce the fibrosis promoter TGF-β1 to improve BPH. Studies have shown that *Salmonella* infection may elevate ROS and glutathione levels, and that engineered *Salmonella* YB1 can reduce GPX4 expression in glioma cells, inducing ferroptosis [37, 38]. Our findings align with some of these observations. *S.e*-LPS can significantly promote ferroptosis in prostate cells, increase ROS and MDA production, and exacerbate lipid peroxidation in prostate cells. Recent research has demonstrated a significant association between m^6^A modification and ferroptosis through the regulation of gene expression. Zou et al. [39] reported that m^6^A-mediated regulation of fibroblast growth factor receptor (FGFR) 4 alleviated ferroptosis in patients with refractory HER2-positive breast cancer. Our prior investigation also revealed that m^6^A modification influences the stability of LINC00641, thereby impacting ferroptosis [40]. *S.e*-LPS significantly reduced the level of m^6^A, increased the mRNA and protein levels of m^6^A eraser ALKBH5, and regulated *GPX4* mRNA stability via ALKBH5, thereby promoting ferroptosis and fibrosis in prostate cells. Our results suggest that m^6^A modification may serve as a bridge for microbial-mediated ferroptosis in prostate cells.

Evidence indicates that ferroptosis promotes tissue fibrosis and plays an important role in conditions such as liver cirrhosis, renal fibrosis, cardiomyopathy, idiopathic pulmonary fibrosis, and other fibrotic diseases. Ferroptosis inhibitors exhibit anti-fibrotic properties [41]. Ferroptosis contributes to the progression of fibrosis through mechanisms such as mitochondrial damage, membrane densification, and fibroblast activation [42]. Additionally, ferroptosis exacerbates fibrosis by inducing oxidative stress, which promotes EMT and ECM deposition [43]. Ferroptosis regulates fibrotic progression across multiple organs via distinct pathways [44], and the inhibition of ferroptosis has been shown to effectively mitigate prostate inflammation and fibrosis [45]. However, the involvement of ferroptosis in the development of BPH and microbial-induced prostate fibrosis has not been investigated. Lisa et al. [46] demonstrated that the bacterial signal transduction adaptor MyD88 is crucial for driving COX2 signaling, which plays an important role in intestinal fibrosis during *Salmonella*-induced enterocolitis. GPX4, a classical inhibitor of ferroptosis, when down-regulated, can aggravate pulmonary fibrosis, liver fibrosis, and other fibrotic diseases by regulating autophagy, oxidative stress, and other pathways [47]. Matrix metalloproteinases (MMPs) and TIMPs are critical factors in regulating ECM degradation. While MMPs play a role in degrading ECM during fibrosis, their activity can also compromise the structural integrity of normal ECM, leading to tissue injury and exacerbation of fibrotic processes [48]. In this study, *S.e*-LPS was found to induce a time-dependent upregulation of MMP2 and MMP9 in prostate cells, accompanied by a concomitant suppression of TIMP1 and TIMP3 expression. Notably, the application of Ferrostatin-1 and the knockdown of *ALKBH5* were able to reverse these LPS-driven alterations, mechanistically linking ferroptosis and m^6^A RNA modification to fibrotic pathways. This observation aligns with evidence in other diseases, where ALKBH5 modulates MMP expression and ECM remodeling through its m^6^A demethylase activity [49, 50]. Our findings demonstrated that in prostate tissues with a high abundance of *S. enterica*, there was a significant increase in collagen fibers and a decrease in GPX4 expression. Furthermore, the inhibition of ferroptosis was shown to attenuate *S.e-*LPS-induced fibrosis in prostate cells.

This study also has some limitations. Our clinical sample size was limited, and microbes vary greatly among individuals. Although in vitro and animal experiments have elucidated the impact of *S. enterica* on prostate fibrosis, the epidemiological data regarding the role of *Salmonella* in the severity of BPH and prostate fibrosis remain insufficient. Moreover, future studies with larger sample sizes may reveal the presence of other microorganisms that play a significant role. Therefore, there is a pressing need for longitudinal research with larger sample sizes in the future. In addition, this study did not fully address whether other virulence factors, such as bacterial flagella or specific genes, contribute to the involvement of *S. enterica* in BPH.

## Conclusions

*S. enterica* is enriched in high-fibrosis prostatic tissue and has been demonstrated through cellular and animal experiments to induce prostatic fibrosis. *S. enterica* can promote ferroptosis and fibrosis in prostate cells by regulating the stability of *GPX4* mRNA via ALKBH5-mediated m^6^A modification. These findings suggest that *S. enterica* induces fibrosis in the prostate through the ALKBH5-m^6^A-GPX4-mediated ferroptosis pathway, potentially offering insights into the development of novel drug targets and personalized prevention and treatment strategies for BPH from microbial and epigenetic perspectives.

## Supplementary Information

**Additional file 1. Table S1** RNA oligonucleotides sequences used in this study. **Table S2** Antibodies used for immunofluorescence (IF) and immunohistochemistry staining (IHC). **Table S3** Primers and RNA oligonucleotides sequences used in this study. **Table S4** Antibodies used for Western blotting analysis. **Table S5** Clinical data of tissue samples from patients with BPH. **Fig. S1*** S.e*-LPS promotes cell proliferation and lipid peroxidation.

## Data Availability

The 16S rDNA microbiome sequencing data supporting the findings of this study are publicly accessible in NCBI BioProject at: https://www.ncbi.nlm.nih.gov/bioproject, reference number: PRJNA1204186. Additional data utilized in this study are provided within the article.

## Abbreviations

ALKBH5: AlkB homolog 5
BPH: Benign prostatic hyperplasiah
CF: Collagen fibers
ECM: Extracellular matrix
EMT: Epithelial-mesenchymal transition
FTO: Fat mass and obesity-associated protein
FGFR4: Fibroblast growth factor receptor 4
GPX4: Glutathione peroxidase 4
HF: High-fibrosis
IF: Immunofluorescence
IHC: Immunohistochemistry
KEGG: Kyoto encyclopedia of genes and genomes
LF: Low-fibrosis
m^6^A: N^6^-methyladenosine
METTL3: Methyltransferase-like 3
MMP: Matrix metalloproteinase
OTUs: Operational taxonomic units
PCoA: Principal coordinate analysis
PLS-DA: Partial least squares discriminant analysis
PICRUSt2: Phylogenetic investigation of communities by reconstruction of unobserved states 2
ROS: Reactive oxygen species
RSL3: Ras-selective lethal 3
*S. enterica*: *Salmonella enterica*
*S.e*-LPS: Lipopolysaccharide of *Salmonella enterica*
SM: Smooth muscle
SRAMP: Sequence-based RNA adenosine methylation site predictor
T-BPH: Testosterone-induced BPH
TURP: Transurethral resection of the prostate
