# Supplementary material for: Salmonella enterica mediated epigenetic promotion of fibrosis is a novel factor in benign prostatic hyperplasia
Source: Mil Med Res. 2025 May 29;12:24. doi: 10.1186/s40779-025-00614-2 (PMC12121288; doi:10.1186/s40779-025-00614-2)
Supplement: Supplementary file 1 — Additional file 1. Table S1 RNA oligonucleotides sequences used in this study. Table S2 Antibodies used for immunofluorescence (IF) and immunohistochemistry staining (IHC). Table S3 Primers and RNA oligonucleotides sequences used in this study. Table S4 Antibodies used for Western blotting analysis. Table S5 Clinical data of tissue samples from patients with BPH. Fig. S1 S.e-LPS promotes cell proliferation and lipid peroxidation. [file 40779_2025_614_MOESM1_ESM.pdf]

Methods

Cell proliferation assay

WPMY-1 and BPH-1 cells (2 × 10^3 cells/well) were seeded into a 96-well plate, and cell proliferation was assessed using the cell counting kit-8 (CCK-8, Dojindo Laboratories, Kumamoto, Japan). All experiments were performed in triplicate. Proliferation curves were generated based on absorbance measurements taken at specified time intervals. To assess cellular sensitivity to the ferroptosis inducer RSL3, cells (4 × 10^3) were seeded into a 96-well plate and treated with *S.e*-LPS for 24 h, followed by exposure to various concentrations of the drug for an additional 24 h. CCK-8 assay was performed to measure cell viability at the indicated time points, and IC_{50} values were calculated using GraphPad Prism.

Cell apoptosis, cycle assays

WPMY-1 and BPH-1 cells (1.5 × 10^5 cells/well) in a 6-well plate were treated with the indicated concentrations of *S.e*-LPS and then incubated for 24 h. Cells were collected after washing with phosphate buffered saline (PBS) three times and gently mixed with Annexin V-FITC and propidium iodide staining solution by using the Apoptosis Detection Kit (LiankeBio, Hangzhou, China). It was incubated at room temperature for 15 min, and they were detected by flow cytometry (NovoCyte 3000, Agilent Technologies, CA, USA) after 15 min of incubation. For cell cycle distribution analysis, cells were collected and detected by the Cell Cycle Staining Kit (LiankeBio, Hangzhou, China) according to the manufacturer’s instructions. Then the cells were detected by flow cytometry.

Cell contraction assays

WPMY-1 cells were suspended (3.0 × 10^6 cells/ml) in a cell contraction matrix (Cell Biolabs, San Diego, CA, USA). This collagen-cell mixture was dispensed in 0.5 ml aliquots into 24-well culture plates, and the mixture was polymerized at 37 °C for 1 h. Immediately after polymerization, 1 ml of culture medium with *S.e*-LPS (1 μg/ml) or PBS was added to each well. After 72 h incubation, the collagen gels were photographed, and the surface area of the gels was measured.

Reactive oxygen species (ROS) and lipid peroxidation detection and measurement

The presence of ROS was measured by ROS Assay Kit (Beyotime, Shanghai, China), while lipid peroxidation was measured by BODIPY™ 581/591 C11 Assay Kit (Thermo Fisher, Shanghai, China).

Following 24 h of various treatments, 10 μmol/L DCFH-DA was introduced into the medium for ROS detection, and 5 μmol/L was added for lipid peroxidation assessment. Then incubated together for 30 min at 37 °C. Post-incubation, the samples were washed three times with PBS, and then cells were collected and analyzed by flow cytometry. The FITC channel signal was monitored and quantified. A minimum of 1×10^4 cells were analyzed per sample, with each experiment conducted independently at least three times.

Immunofluorescence cell staining

WPMY-1 and BPH-1 cells were cultured on a sterile confocal petri dish (Biosharp, Anhui, China), subsequently fixed in methanol for 15 min, permeabilized with 0.1% Triton X-100, and blocked with 1% bovine serum albumin (BSA) for 30 min. The cells were then incubated overnight at 4 °C with either rabbit anti-m^6A (Epigentek, USA) or rabbit anti-ALKBH5 (Abcam, USA) antibodies. After washing three times, the cells were probed with Alexa-Fluor-555-conjugated goat anti-rabbit-IgG (Abcam) for 1 h at room temperature, followed by nuclear counterstaining with 4',6-diamidino-2-phenylindole (DAPI) (Beyotime, Shanghai, China). Observations were conducted using confocal fluorescence microscopy (Leica, Germany).

Transmission electron microscopy of mitochondrial structures

For detecting the morphological changes associated with ferroptosis, samples were fixed in 3% phosphate-glutaraldehyde at 4 °C for 4 h, washed with 0.1 mol/L PBS (pH 7.4), and embedded in 1% agarose. Post-fixation with 1% osmium tetroxide was performed for 2 h, followed by gradient ethanol dehydration (30 – 100%) and acetone substitution. Infiltration was achieved using 812 embedding resins mixed with acetone at 37 °C, followed by polymerization in pure resin at 60 °C for 48 h. Ultrathin sections (60 nm) were collected on copper grids, stained with 2% uranyl acetate and 2.6% lead citrate, and dried for analysis. Cells were observed in transmission electron microscopy (TEM) by Hitachi (HT7700 Exalens).

Table S1 RNA oligonucleotides sequences used in this study

**Gene**	**Sequence (5'-3')**
shALKBH5#1	GCGCCGTCATCAACGACTA
shALKBH5#2	CTGCGCAACAAGTACTTCT
shALKBH5 (rat)	CGGCCTCAGGACATCAAAGAA

Table S2 Antibodies used for immunofluorescence (IF) and immunohistochemistry staining (IHC)

**Antibodies**	**Source species**	**Dilution ratio**	**Supply**
Primary antibody			
N^6-methyladenosine (m^6A) Antibody	Rabbit	1:500 (IF)	Epigentek, A-1801-100
ALKBH5	Rabbit	1:200 (IF)	Abcam, ab195377
GPX4	Mouse	1:500 (IHC)	Proteintech, 67763-1-Ig
Collagen I	Rabbit	1:1000 (IHC)	Servicebio, GB115707
Salmonella enterica serotype enteritidis LPS antibody	Mouse	1:50 (IF)	Sigma SAB4200882
Secondary antibody			
Donkey Anti-Rabbit IgG H&L (Alexa Fluor® 647)	Donkey	1:1000 (IF)	Abcam, ab150075
Goat anti-mouse-IgG-HRP	Goat	1:1000 (IHC)	Abcam, ab6789
Goat anti-rabbit-IgG-HRP	Goat	1:2000 (IHC)	Abcam, ab205718

ALKBH5 AlkB homolog 5, *GPX4* glutathione peroxidase 4, *LPS* lipopolysaccharide, *HRP* horseradish peroxidase

Table S3 Primers and RNA oligonucleotide sequences used in this study

Gene	Forward primer (5' – 3')	Reverse primer (5' – 3')
METTL3	TTAGCCTTCGGGGTGTCCG	TAGATCCAAGTGCCCCGAGT
METTL4	CTTGGTCTGTGGAGGTAGTTGC	CCAGTATAAGACCTTCGTAGGGC
METTL5	GCCCAAGCTACTTCTGGAACAG	CCGATGCTAAGTACTCCACAACC
METTL14	CTGAAAGTGCCGACAGCATTGG	CTCTCCTTCATCCAGATACTTACG
METTL16	TGGAGCAACCTTGAATGGCTGG	CCATCAGGAGTGTCTTCTGTGG
WTAP	GCAACAACAGCAGGAGTCTGCA	CTGCTGGACTTGCTTGAGGTAC
KIAA1429	TGACCTTGCCTCACCAACTGCA	AGCAACCTGGTGGTTTGGCTAG
RBM15	GATTCCTCTCCACACCTGACTTC	TGTTGCCAGAGACGAAGTGGAG
RBM15B	TGGTAACCTGGACCACAGCGTA	GGTTCTGGAACTTGAGGAAGGC
ZC3H13	CGGACAGTGATGCCTACAACAG	TCTGTGAGGTGCGAGGGACTAA
CBLL1	AACAGGATGCCTGCAAAGGCTC	GGTGTCCAGGAAATCTTCGCTG
ZCCHC4	CTTCCAGATGCTGGATTACCAGG	CGGAATGTTGGTGAAAATACGCAC
PCIF1	CTCTGCCTTTGAGAGGTTCCTG	AGCACTCGAAGCTGACGCCAAA
ALKBH1	TTACTACCGCCTGGACTCCACA	CCTCATCCCTTTGAAGACCACC
ALKBH5	CCAGCTATGCTTCAGATCGCCT	GGTTCTCTTCCTTGTCCATCTCC
FTO	CCAGAACCTGAGGAGAGAATGG	CGATGTCTGTGAGGTCAAACGG
YTHDF1	CAAGCACACAACCTCCATCTTCG	GTAAGAAACTGGTTCGCCCTCAT
YTHDF2	TAGCCAGCTACAAGCACACCAC	CAACCGTTGCTGCAGTCTGTGT
YTHDF3	GCTACTTTCAAGCATACCACCTC	ACAGGACATCTTCATACGGTTATTG
YTHDC1	TCAGGAGTTCGCCGAGATGTGT	AGGATGGTGTGGAGGTTGTTCC
YTHDC2	GAAAGCTCCTGAACCTCCACCA	GGTTCTACTGGCAAGTCAGCCA
ELAVL1	TGTTCTCTCGGTTTGGGCGGAT	TCTTCTGCCTCCGACCGTTTGT
FMR1	CAAAGGACAGCATCGCTAATGCC	GCTCCAATCTGTCGCAACTGCT
EIF3A	CGTAATGCGACTCAAAGCTGCAC	ACGCTGCCTTTTCCGTTCTTCC
G3BP1	AGCCTGTTCAGAAAGTCCTTAGC	CGAAGGCGATTATCTCGTCGGT
G3BP2	GAGCTGAAACCACAAGTGGAGG	GGTCACTGAAGCCCAGGAGAAA
HNRNPA2B1	CAGCAACCTTCTAACTACGGTCC	CACTGCCTCCTGGACCATAGTT
PRRC2A	CTTCTACCCTCCTGGTGTGCAT	CGTTCCCGTAACATAGCAGGTG
IGF2BP1	CTTTGTAGGGCGTCTCATTGGC	CCTTCACAGTGATGGTCCTCTC

Gene	Forward primer (5' – 3')	Reverse primer (5' – 3')
IGF2BP2	GTTGGTGCCATCATCGGAAAGG	TGGATGGTGACAGGCTTCTCTG
IGF2BP3	TCGTGACCAGACACCTGATGAG	GGTGCTGCTTTACCTGAGTCAG
HNRNPC	TGGGCTGCTCTGTTCATAAGGG	CTCGGTTCACTTTTGGCTCTGC
GPX4	ACAAGAACGGCTGCGTGGTGAA	GCCACACACTTGTGGAGCTAGA
SLC7A11	TCCTGCTTTGGCTCCATGAACG	AGAGGAGTGTGCTTGCGGACAT
ACSL4	GCTACTTGCCTTTGGCTCATGTGC	GTGTGGGCTTCAGTACAGTACAGTCTCC
GPX4-1	CGCCGCGATGAGCCT	GTGACGATGCACACGAAGC
GPX4-2	CACCGTCTCTCCACAGTTCC	ACGCTGGATTTTCGGGTCTG
GPX4-3	GGACCTGCCCCACTATTTCTA	TTTATTCCCACAAGGTAGCCAG

METTL3 methyltransferase like 3, *WTAP* Wilms tumor 1-associated protein, *RBM15* RNA binding motif protein 15, *ZC3H13* Zinc finger CCCH-type containing 13, *CBLL1* Casitas B-lineage lymphoma proto-oncogene-like 1, *ZCCHC4* Zinc finger, CCHC-type containing 4, *PCIF1* phosphorylated CTD interacting factor 1, *ALKBH1* Alk B homolog 1, *FTO* fat mass and obesity-associated protein, *YTHDF3* YTH domain family member 3, *ELAVL1* ELAV-like RNA binding protein 1, *FMR1* fragile X mental retardation 1, *EIF3A* eukaryotic translation initiation factor 3 subunit A, *G3BP1* GTPase activating protein binding protein 1, *HNRNPA2B1* heterogeneous nuclear ribonucleoprotein A2/B1, *PRRC2A* proline-rich coiled-coil 2A, *IGF2BP3* insulin-like growth factor 2 mRNA binding protein 3, *HNRNPC* heterogeneous nuclear ribonucleoprotein C, *GPX4* glutathione peroxidase 4, *SLC7A11* solute carrier family 7 member 11, *ACSL4* Acyl-coa synthetase long-chain family member 4

Table S4 Antibodies used for Western blotting analysis

Antibodies	Source species	Dilution ratio	Supply
Primary antibody			
ALKBH5	Rabbit	1:1000	Abcam, ab195377
GPX4	Rabbit	1:1000	Abclonal, A11243
SLC7A11	Rabbit	1:1000	Abclonal, A2413
ACSL4	Rabbit	1:1000	Proteintech, 22401-1-AP
α-SMA	Rabbit	1:10,000	abcam, ab124964
N-cadherin	Rabbit	1:2000	Proteintech, 22018-1-AP
Vimentin	Rabbit	1:20000	Abclonal, A19607
Collagen I	Rabbit	1:1000	Abcam, ab138492
MMP2	Rabbit	1:1000	Abcam, ab97779
MMP9	Rabbit	1:1000	Abcam, ab76003
TIMP1	Rabbit	1:1000	Abcam, ab211926
TIMP3	Rabbit	1:1000	Proteintech, 10858-1-AP
GAPDH	Rabbit	1:10,000	Abcam, ab181602
Secondary antibody			
Anti-rabbit-IgG (H + L)-HRP	Goat	1:10,000	Beyotime Biotechnology, A0208

ALKBH5 AlkB homolog 5, *GPX4* glutathione peroxidase 4, *SLC7A11* solute carrier family 7 member 11, *ACSL4* acyl-coa synthetase long-chain family member 4, *α-SMA* α-smooth muscle actin, *MMP* matrix metalloproteinase, *TIMP1* tissue inhibitor of metalloproteinases 1, *GAPDH* glyceraldehyde-3-phosphate dehydrogenase

Table S5 Clinical data of tissue samples from patients with BPH

Parameters	LF (*n* = 9)	HF (*n* = 10)	*P*-value
Age (year, mean ± SD)	71.2 ± 7.6	68.9 ± 8.1	0.529
Volume (ml, mean ± SD)	50.93 ± 16.29	67.87 ± 26.65	0.118
IPSS (score, mean ± SD)	21.67 ± 6.44	29.30 ± 6.50	0.020
tPSA (μg/L, mean ± SD)	5.93 ± 4.58	11.45 ± 10.46	0.163
fPSA [μg/L, M (Q_1, Q_3)]	0.74 (0.62, 1.16)	1.41 (0.64, 2.78)	0.360
fPSA/tPSA (mean ± SD)	0.21 ± 0.07	0.22 ± 0.10	0.869
PSA density (ng/ml^2, mean ± SD)	0.12 ± 0.10	0.16 ± 0.11	0.478
BMI (kg/m^2, mean ± SD)	21.63 ± 4.00	23.53 ± 2.34	0.258
Hypertension [*n* (%)]	3 (33.3)	4 (40.0)	1

BPH benign prostatic hyperplasia, *IPSS* International Prostate Symptom Score, PSA prostate-specific antigen, *tPSA* total PSA, *fPSA* free PSA, *BMI* body mass index, *LF* low-fibrosis, *HF* high-fibrosis

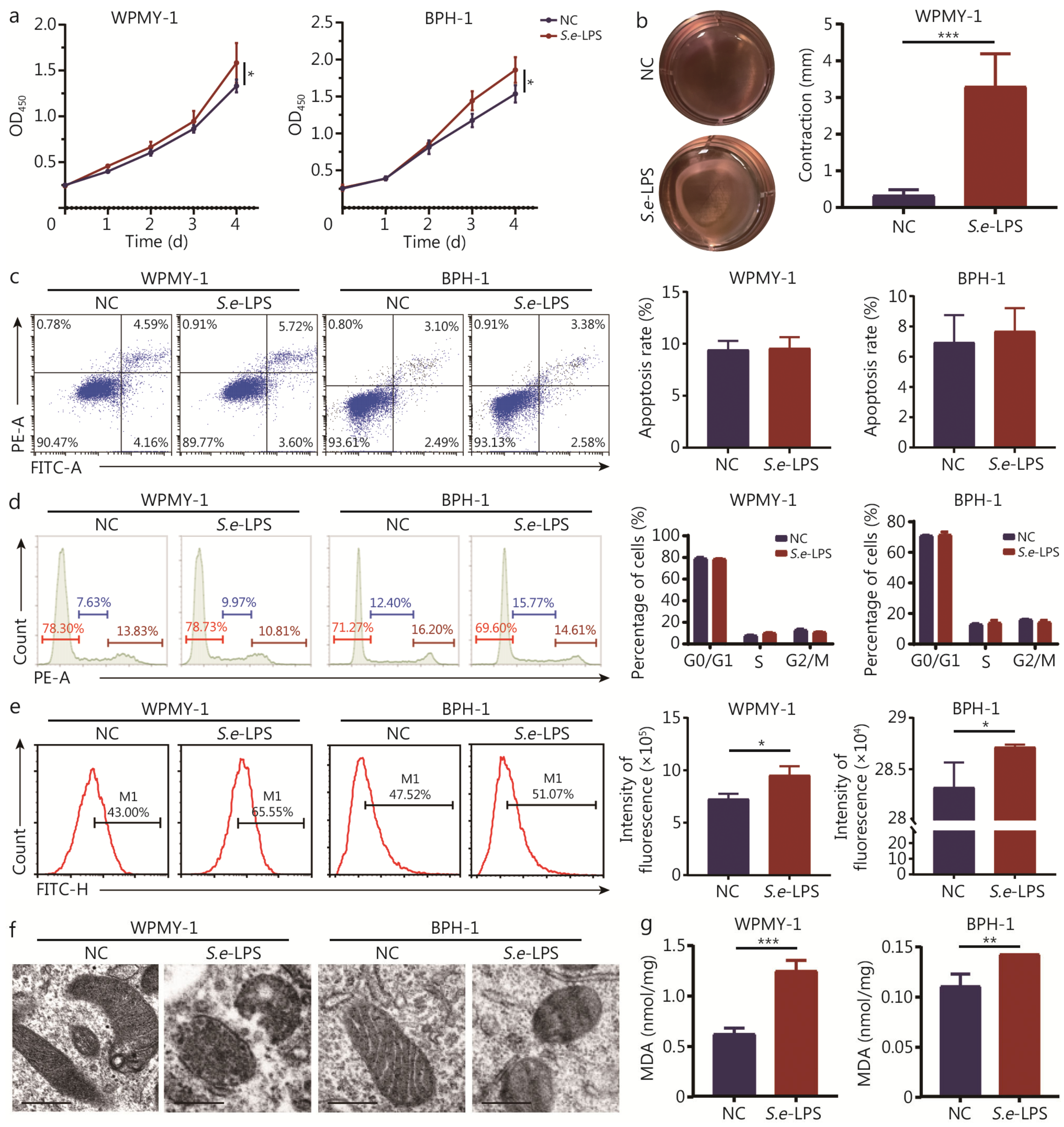

Fig. S1 *S.e*-LPS promotes cell proliferation and lipid peroxidation. **a** Cell proliferation curves were detected by CCK-8 assays after treatment with *S.e*-LPS in WPMY-1 and BPH-1 cells. **b** Capacity of cell contraction was detected by cell contraction assay after treatment with *S.e*-LPS in WPMY-1 cells. **c** Flow cytometry apoptotic representative images of WPMY-1 and BPH-1 cells treated with *S.e*-LPS for 24 h. **d** Flow cytometry representative images and quantitative analyses of the cell cycle of WPMY-1 and BPH-1 cells treated with *S.e*-LPS for 24 h. **e** Flow representative images and quantitative analysis of ROS after treatment with *S.e*-LPS for 24 h in WPMY-1 and BPH-1 cells. **f** Representative images of transmission electron microscopy of WPMY-1 and BPH-1 cells. Scale bar = 200 nm. **g** Results of malondialdehyde (MDA) in WPMY-1 and BPH-1 cells after treating with *S.e*-

LPS. $^{*}P < 0.05$, $^{**}P < 0.01$, $^{***}P < 0.001$. *S.e*-LPS lipopolysaccharide of *Salmonella enterica*, MDA malondialdehyde
